# Revealing the secret life of pre-implantation embryos by time-lapse monitoring: A review

**Published:** 2017-05

**Authors:** Azita Faramarzi, Mohammad Ali Khalili, Giulietta Micara, Azam Agha-Rahimi

**Affiliations:** 1 *Research and Clinical Center for Infertility, Yazd Reproductive Sciences Institute, Shahid Sadoughi University of Medical Sciences, Yazd, Iran.*; 2 *Department of Anatomical Sciences and Biology, Faculty of Medicine, Kermanshah University of Medical Sciences, Kermanshah, Iran.*; 3 *Department of Obstetrics and Gynecology, UOC OGC03 Infertility and IVF University of Rome La Sapienza, Viale Regina Elena 324-00161 Rome*

**Keywords:** Embryo selection, Kinetic, Assisted reproductive technology, Time-lapse

## Abstract

High implantation success following in vitro fertilization cycles are achieved via the transfer of embryos with the highest developmental competence. Multiple pregnancies as a result of the transfer of several embryos per cycle accompany with various complication. Thus, single-embryo transfer (SET) is the preferred practice in assisted reproductive technique (ART) treatment. In order to improve the pregnancy rate for SET, embryologists need reliable biomarkers to aid their selection of embryos with the highest developmental potential. Time-lapse technology is a noninvasive alternative conventional microscopic assessment. It provides uninterrupted and continues the survey of embryo development to transfer day. Today, there are four time-lapse systems that are commercially available for ART centers. In world and Iran, the first time lapse babies were born in 2010 and 2015, respectively, conceived by SET. Here, we review the use of time-lapse monitoring in the observation of embryogenesis as well as its role in SET. Although, the findings from our review support common use of time-lapse monitoring in ART centers; but, future large studies assessing this system in well-designed trials are necessary.

## Introduction

In vitro fertilization (IVF) as one of assisted reproductive technique (ART) methods is used in infertility centers for conceiving infertile couples. In vitro culture of human embryo technique has improved considerably in recent years. New incubators and medium have been bettered which provide embryo development to blastocyst (1). In spite of this, best embryo selection for transfer is the main challenge in ART clinics. The failure to suitably know the embryos with highest developmental competence can lead to failed ART cycles. Grading categorical based on morphology is the preferred method for recognizing embryo developmental potential, despite promising other methods (2). 

However, embryologists still depend on daily discrete assessments of embryo development based on cell count on cleavage days 2 or 3, the extent of blastulation as well as the quality of the inner cell mass or trophectoderm in blastocysts on day 5, the degree of fragmentation, the rate of nucleation, and symmetry in cleavage embryos (1). Unfortunately, conventional morphological techniques for evaluating embryo development have been preliminary and have not yet increased desirable results of implantation and pregnancy (3). TLM has first used three decades ago for the study of the developmental progression of bovine embryos (4, 5). Recent interest in TLM for assessment of clinical embryos was engendered following numerous studies investigating the potential benefit of multi-day scoring of embryos for selection of the most robust embryo(s) for ET (6). 

Despite conflicting findings, it is assumed that compared with static observations, frequently captured images will provide substantial information regarding the association between morphological development and embryo viability and a more developmental kinetics (1). Motato *et al* showed that main advancement in the selection of the best embryos with TLM is recognition of the embryos with high implantation competence (7). However, the comparison of TLM with conventional embryo incubation was reviewed by Cochrane in 2015, comparing clinical pregnancy, live birth, miscarriage, and stillbirth rates. The data showed that there were no significant differences in any of these clinical outcomes (8). Some data extraction was thus done on 13 summarized papers in table I.

In this review, an overview of the current literature concerning the use of this technology in regards to the best embryo selection with a possible increase of implantation rate in ART cycles is discussed. A literature search in PubMed, Scopus, Ovid MEDLINE, Web of Science, and Science Citation Index was done to identify studies that evaluated the embryo selection based on morphokinetics by TLM system.

**Why should be chosen the best embryo?**

In spite of ASRM guidelines to reduce the number of embryos transferred, many centers still transfer 2-3 embryos despite the increase in the risk of multiple pregnancies (9). Multiple pregnancies are unfavorable due to the poor neonatal outcome, maternal complications, long-term developmental problems and high costs. Multiple pregnancies are associated with increased rates of stillbirths, neonatal deaths, and infant mortality. Multiple gestations have an increased morbidity rate due to complications associated with the increased risk of prematurity, low birth weight. Multiples also have a compromised long-term outcome, including an increased risk of long-term medical and developmental problems, such as learning problems, cerebral palsy, as well as adult health risks.

Pregnancy-induced maternal complications, particularly hypertensive disorders, are more common in association with multiple pregnancies (10). It also entails an increased risk for intrapartum complications as a consequence of uterine atony and malpresentation, resulting in high incidences of cesarean sections and postpartum hemorrhage. In addition, the financial consequences of multiple pregnancies are substantial for both parents and healthcare providers (9). 

SET is now considered as the preferred practice in IVF cycles to reduce the risk of adverse outcomes associated with multiple gestations. However, in order to improve the pregnancy outcomes for SET, embryologists depends on biomarkers to aid their selection of the embryos with the highest developmental potential (11). In the SET program, that includes the transfer of a single fresh embryo in selected women with good prognosis (<35 yrs of age), pursued by one or more frozen-warmed embryo transfer cycle(s) as required, has diminished multiple pregnancy rates, while keeping passable live birth rates (12). Over the past decade, SET utilization has been an increment in assisted conception facilities. However, there are challenges to increase use of SET as standard ET program. It involves fine embryo selection, patient education and provider, and successful cryopreservation (13).

**Best embryo selection procedure**

As SET becomes increasingly applied in many ART practices, the challenge of identifying the embryo with the highest developmental competence becomes crucial. Therefore, there have been investigations in search of additional viability markers to supplement current criteria for selection, such as aneuploidy screening, O_2_ respiration, metabolic profiling and gene expression analysis (14). Although, these methods are surely promising, but grading systems based on embryo morphology still remain the preferred method for assessing embryonic competence (15). 

However, these morphological assessments are generally limited to once a day checking, since repeated removal of embryos from the incubator for observation resulted in undesired temperature and pH fluctuations in the culture dish. Embryo development is a dynamic event and static observations of their growth can be limiting in their ability to discern differences between embryos at similar cell stages (16, 17). Blastocyst transfer is one of the successful approaches for SET, but it may increase neonatal complications, such as of preterm birth, low birth weight, respiratory diagnoses and low APGAR score, owing to the extended culture condition (18, 19). Therefore, identifying cleaving embryos that will develop to the blastocyst stage is necessary (20). The early prediction that which embryo may reach to the blastocyst before day 3 of the development will let an earlier transfer to be done. Hence, the need for longer culture to blastocyst is well eliminated, which is possible by using TLM (21).

**Modern embryo survey: morphokinetics (using TLM)**

The new era for embryo quality evaluation takes a different method: dynamic morphokinetics. It is the result of the application of TLM in the ART centers which combines morphokinetics and morphology of embryo development. The precise timing of specific events, such as pronuclear formation, syngamy appearance, early cleavage events, cell cycle intervals, the synchronicity of cell division and initiation of blastulation are indicators of an embryo’s developmental potential. The ability to continuously monitor an embryo’s progression certainly aids in selecting the best embryo/s for uterine transfer (16, 17). 

**Correlation of morphokinetics and aneuploidy in embryos**

Absence or presence of an additional chromosome which causes to divergence from 46 chromosomes in humans is defined as aneuploidy. Embryo aneuploidy results in implantation failure and increases in miscarriage rates (22). The conventional procedures to distinguish aneuploidy in human embryos are preimplantation genetic screening (PGS) and preimplantation genetic diagnosis (PGD), which are expensive technologies and not routinely accessible. Also, biopsy of one or more cells of embryos is needed for analysis (23). 

TLM by using certain morphokinetic parameters may distinguish aneuploidy Thus, TLM can be used to select the euploid embryos noninvasively as an alternative to PGS (24). Recently, Chawla and associates assessed the morphokinetic parameters such as timings of the extrusion second PB, pronuclei appearance and fading, division timing and second and third cleavages duration of 460 embryos in order to discriminate between euploid and aneuploid embryos. Their results showed that morphokinetic parameters differed significantly for euploid and aneuploid embryos (25). 

In contrast to this, some studies showed contradictory results, with regards to knowing kinetic parameters that are classified as euploid or aneuploid embryos (26). Although TLM could be a potential selection instrument for women who are not a candidate for PGS, there is still a need for more studies to be done before replacing PGS and/or PGD with TLM (27).

**Time-lapse systems models**

At present, there are four TLM systems which are used in the embryology laboratory, namely Primo Vision, EmbryoScope, Ecso Miri and Eeva systems (Table II). They all require the use of a digital inverted microscope that acquires images of the embryos at preset intervals which are integrated to create live images. The Embryo Scope and Ecso Miri are compact, a self-contained incubator with a built-in camera. While, both Eeva and Primo Vision systems comprise a camera that is placed in a traditional incubator (9, 11, 14, 15, 28). 

Each of the systems uses a different light source and differs in the way the embryos are brought into the field of view (no movement of the embryo culture dish vs. constant movement of the dish). The EmbryoScope, Esco Miri and Primo Vision systems use bright field technology that allows the assessment of both kinetic parameters and morphology of the embryos. While, the dark field technology used with Eeva allows the assessment of kinetic parameters, but provides limited information on the morphological features. 

Although, all systems use an oil overlay on culture microdrops, but differ in the way the embryos are cultured: the Eeva dish and the microwell group culture dish for Primo Vision provide group culturing, in which 12 micro-wells (Eeva) and 9-16 wells (Primo Vision) share a common 50-120 μl volume of medium. In contrast, the EmbryoScope provides an individual culture set-up, in which the culture dish has 12 wells, each holding 20-25 μl of culture medium (1, 9, 28). 

**The benefits and safety of time-lapse technology**

The first advantage of TLM is that the embryos are retained in a low disturbed environment during development, as they are not subjected to alteration in gas, pH, or temperature changes, or to the movements that are done daily for embryo assessment under conventional conditions. The second advantage correlated to the extra developmental morphokinetic that are obtained as compared with conventional assessment at different time points. Human embryos display discontinuously morphologic features that are commonly used during conventional morphological grading system (1, 8).

Key morphokinetics parameters (Table III) from TLM for prognostication of blastocyst formation, aneuploidy and implantation have been surveyed during recent years (25, 29, 30-35). Multiple studies have reported that apply of determined morphokinetic parameters is correlated with betters prediction of embryo selection. Furthermore, increased implantation and pregnancy rates have been shown which has encouraged various IVF clinics to purchase TLM system (8). In spite of increased studies showing models and algorithms for selecting the best embryos, it is presumably which timing of development is distinguished by the health of embryo. Though, further culture condition variables, intrinsic patient characteristics, as well as the type of ovarian stimulation protocol and use of ICSI, could play important roles (1). 

Also, TLM has improved the knowledge on the mechanism of fertilization and early preimplantation embryo development. Beneficial data for embryo selection is obtained by TLM to pursue the dynamic pattern of embryo development. In addition, TLM lets observation of specific time point to be related to the capacity of embryo development and implantation. Moreover, TLM prepares a united monitoring including a safe culture environment that determines critical event of embryo development (36, 37). Before applying TLM in clinical centers, the safety of it is an important issue to be considered. TLM entails serial light exposure. It has been reported that vast exposure to light may be deleterious to the embryo, and particularly that exposure of wavelength light ought to be minimized (15). However, it has been reported there was no detrimental effect of obtaining images of a microscope on the development of human embryos (38).

Down intensity red light (635 nm) from a single light-emitting diode with low illumination times of 30 ms per image to short embryo exposure to light and to elude harming short wavelength light is used in Embryo Scope. Also, evaluations were made on conventional microscope applied in IVF centers. The time of light exposure in the time-lapse system during 3 days for a total of 1420 images was 57s, compared with a higher light exposure time of 167s reported for a conventional IVF system (38).

**Table I:** Association of TLM parameters with human embryo development in 15 eligible studies retrieved from electronic database search and reference list review

**Authors**	**Embryos no./ Patients no.**	**Start of imaging/ the time between image acquisition**	**Comments**
Cetinkaya *et al* (2015) (43)	3,354 /626	time of insemination/ 20 min	Cleavage relative timings were better indicators of blastocyst formation and quality compared to absolute time-points
Storr *et al* (2015) (30)	380/108	time of ICSI/ 7 to 20 min	Eight significant predictive parameters of a top quality blastocyst were known: s3, t6, t7, t8, tM, tSB, tB and tEB *
Siristatidis *et al* (2015) (44)	Not reported/239	Time of ICSI/ 10 min	Early embryo morphokinetics parameters were associated with the subfertile patients characteristics
Almagor *et al* (2015) (45)	Not reported/253	Time of ICSI/ Not reported	Irregular cleavage embryos that are prevalent in younger women may have implantation potential and live birth.
Motato *et al* (2015) (7)	7,483/not reported	Time of ICSI/ 15 min	Morphokinetics parameters including tM t8-t5 interval, tEB could predict blastocyst formation and implantation **
Sunddvall *et al* (2015) (46)	1388/249	Time of entry/ 20 min	Developmental timings in PCOS were not linked to live birth ***
Wdowiak *et al* (2015) (47)	Not reported/165	Time of ICSI/ 10 min	Higher SDF levels could be slow down morphokinetic parameters, and might be decreasing of pregnancy rate ****
Wu *et al* (2016) (48)	212/109	After PNA/ Not reported	The use of morphokinetic parameters to select embryo improved implantation and live birth rates
Adamson *et al* (2016) (49)	Not reported/319	After PNA/ Not reported	The use of combined conventional morphology and morphokinetics survey improved implantation rate
Mizobe *et al* (2016) (50).	791/164	Time of entry/ Not reported	Blastocyst transfers that derived from faster first and second cleavage embryos, improved pregnancy rate
Goodman *et al* (2016) (51)	Not reported/235	Time of entry/ 10 min	The use of morphokinetics was not improving ART outcomes, significantly. Although, it associated with blastocyst implantation rates
Liu *et al* (2016) (29)	Not reported/265	Time of insemination/ 10 min	Qualitative and quantitative de-selection proposed model predicted implantation
Nogales *et al* (2017) (52)	485/112	Time of ICSI/ 15 min	Chromosome aneuploidy affects embryo morphokinetics. TLM was useful to know discarded embryos

* S3: third synchronization, t6: time to 6 cells, t7: time to 7 cells, t8: time to 8 cells, tM: time of morula formation, tSB: time to sign of blastulation, tB: time to blastocyst, tEB: time to expanded blastocyst

**: tM: time of morula formation, t8-t5: interval 5 cells to 8 cells, tEB: time to expanded blastocyst

*** : PCOS: poly cystic ovarian syndrome

**** : SDF: Sperm DNA fragmentation

**Table II:** Comparisons of Time lapse Systems Currently Available

**Available time lapse models**	**Embryo scope**	**Evea**	**Primo vision**	**Esco Miri**
Design	Stand-alone integrated incubator/ microscope	Modular in standard incubator	Modular in standard incubator	Stand-alone integrated incubator/ microscope
Maximum no. of embryos monitored per dish	12	12	9 or 16	14
Single or group culture design	single	group	group	Single
Total embryos in one system	72	48	56 or 96	84
Maximum number of focal planes	9	1	11	User defined No limit
Frequency of imaging	From 10 min (2 min with a single focal plane)	5 min	From 5 min	5 min
Imaging/ Illumination	Red LED	Dark field	Green LED	Red LED
Medical device registration	CE medical device class IIa FDA 510 (k)	CE and Canada approved FDA submission pending	System and dish both have CE marking	CE medical device class IIa FDA 510(k) pending

**Table III:** Definition of morphokinetics parameters of embryos

CS2-4	The Cleavage Synchronicity from the 2- to 4-cell
CS4-8	The Cleavage Synchronicity from 4- to 8-cell
DR	The DNA Replication time ratio was defined and calculated by the formula= (t3-t2) / (t5-t3).
T5-PNF	Time from pronuclear fading to 5-cell stage
S2 or P3	duration of 3-cell stage
DC	direct cleavage or where either 2- or 4-cell stage was less than 5 hours
RC	reverse cleavage or where either daughter cells fused after cleavage division or the blastomere failed to divide after karyokinesis,
ICCP	intercellular contact points
P2	duration of 2-cell stage
Pn-t1	Time of pronuclei formation
NEBD	Nuclear envelope break down
t2	Time of cleavage to a 2-cell embryo
t3	Time of cleavage to a 3-cell embryo
t4	Time of cleavage to a 4-cell embryo
t5	Time of cleavage to a 5-cell embryo
t6	Time of cleavage to a 6-cell embryo
t7	Time of cleavage to a 7-cell embryo
t8	Time of cleavage to a 8-cell embryo
tM	Time to full compaction or Morula
tSB	Time to the first signs of blastulation
tB	Time to full blastocyst
tEB	Time to expanded blastocyst
tHB	Time to hatching blastocyst
s1	Time between NEBD and subsequent division to 2 cells
s3	Time between division to 5 cells and subsequent division to 8 cells
t4 interval	Time between division to 4 cells and subsequent division to 5 cells
t5-t2	Time between division to 2 cells and subsequent division to 5 cells
CC2	Duration of the second cell cycle
CC3	Duration of the third cell cycle
S3	time between division from 5 to 8cells
tPNf	Time to pronuclear fading or syngamy

**Limitations of TLM**

Currently, the high expenses of TLM do not let their implementation in many ART centers (39). Although, there are several studies presenting algorithms that may help in the selection of the best embryo, although the timing of early embryo development is mainly determined by the embryo. However, other factors such as the type of insemination, culture condition, type of ovarian stimulation and intrinsic women properties could play a role (1, 40). Also, one of the drawbacks of TLM is that it does not allow rolling of the embryos, causing limited visual observation, especially when a high level of fragmentation exists or blastomeres overlapping other blastomeres (38).

**First time-lapse babies in the world and Iran**

In the year 2010, the analysis of time-lapse records was used to choose a single blastocyst for transfer, which resulted in a singleton pregnancy and first baby conceived with TLM IVF born in Hungary (41). The first Iranian live birth using TLM to select best embryos for transfer was reported in August 2015. A case with tubal factor infertility was admitted to IVF program with normozoospermia. After ovarian hyperstimulation, 6 cumulus oocyte complex (COCs) were retrieved and inseminated with 25,000 progressive sperms/ oocyte. 

Five zygotes were placed individually into the micro wells of equilibrated embryo scope dish for a digital TL microscope (Primo Vision, Vitrolife Co, Sweden) observation, and incubated at 37^o^C, 6% CO_2_, O_2_ 5% and N_2_ 89%. The following early kinetic markers were assessed: time to 2^nd^ polar body (PB) extrusion, pronuclei (PN) appearance, PN fading or syngamy (tPNf), time to 2 cells (c) (t2), 3c (t3), 4c (t4), 5c (t5), 6c (t6), 7c (t7), and 8c (t8). Durations of the second cell cycle (cc2; t3-t2) and the time to complete synchronous divisions s2 (t4-t3) were calculated. Cleavage anomaly was monitored: direct cleavage (single blastomere divided from 1 to 3 cells). The presence of multinucleation, vaculation, and fragmentation were also recorded on day 3, SET took place based on kinetic parameters of the embryos. Clinical pregnancy was confirmed 7 wk after SET (42).

## Conclusion

In general, the practice of multiple embryo transferring not only increase the implantation rates, but also increase the multiple gestations associated with many complications. The ideal state would be high implantation potential SET. In recent years, many efforts have been done to finding suitable approaches to identify the best embryo. Despite of promising other methods, embryo selection based on morphology remains preferred method. However, conventional morphology assessment is subjective and provides limited and discrete data. Recently, the emerging embryo TLM tools have enabled full observation of embryo development. Every change in embryo morphology, from extrusion of the second polar body to the complete blastocyst hatching can be recorded, monitored and assessed. 

In addition, all irregularities and abnormalities of embryo development can be observed, which only monitored by TLM. The application of time lapse microscopy increases embryologist knowledge on embryo morphology and development. Also, it is an effective way for culturing and assessing embryos with minimum disturbing of optimal embryo condition. The data may be used for better selection of embryos for SET, in order to prevent multiple gestations. Continuing monitoring with the use of TLM system lets a more exact identity of embryos that follow likely chromosomally normal. 

Moreover, significant events could be assessed retrospectively at any time before embryo selection for ET. Finally, TLM technology in ART has the great benefit to be a non-invasive method, enable embryo development in very stable condition and correct embryologist decision on selection of embryos for transfer or cryopreservation. Although, the majority of publications have shown optimism regarding the successful application of TL technology in a SET program. Nevertheless, we aspire that large well designed RCTs will define the safety and efficacy of TLM for SET. 

## Conflict of interest

There is no conflict of interests of each author.
